# Dependency profile of the older adult population of a family health unit in northern Portugal

**DOI:** 10.3389/fpubh.2025.1473713

**Published:** 2025-06-10

**Authors:** Mónica Barbosa, Luciana Almeida, Tânia Colaço, Maria Espírito Santo, Rosa Santos, Poliana Martins, Namie Okino Sawada, Teresa Martins

**Affiliations:** ^1^USF Porta do Sol, Unidade Local de Saúde de Matosinhos, Porto, Portugal; ^2^Escola Superior de Enfermagem do Porto, Porto, Portugal; ^3^CINTESIS@RISE, Porto, Portugal; ^4^Enfermagem, Universidade Federal de Alfenas, Minas Gerais, Brazil

**Keywords:** population ageing, functional autonomy, dependency, activities of daily life, older adult health

## Abstract

**Background:**

Population ageing is a global phenomenon, associated with declining birth rates and increased life expectancy, with particularly pronounced effects in European countries such as Portugal. In Portugal, the older population increased significantly, reaching 2.48 million in 2020 and the ageing index is estimated to double by 2080. This study aimed to assess the levels of dependency in basic and instrumental activities of daily living among the older population in a Family Health Unit in northern Portugal to plan effective health interventions.

**Method:**

A cross-sectional study was conducted with a stratified random sampling by age and gender, encompassing 5.7% of a Family Health Unit users aged 65 years or older. The sample included 212 participants, who were assessed using the Barthel Index for Basic Activities of Daily Living (BADL) and the Lawton-Brody Scale for Instrumental Activities of Daily Living (IADL). Data were analysed using descriptive statistics and inferential methods, including t-tests, ANOVA, and logistic regression, to examine associations between functional dependency and sociodemographic variables.

**Results:**

Most participants were independent in BADL (76.89%) and IADL (75.00%), with dependency increasing significantly with age. Women exhibited higher dependency rates than men in BADL (OR = 2.85; *p* = 0.012) and IADL (OR = 2.41; *p* = 0.046). Widowhood was associated with greater dependency in IADL (OR = 2.67; *p* = 0.032). For each additional year of age, the probability of dependency increased by 10% for BADL and 14.8% for IADL.

**Conclusion:**

Functional dependence among older adult individuals in primary healthcare settings highlights the need for targeted interventions to promote autonomy. Gender and age emerged as key predictors of dependency, with women and older adults being particularly vulnerable. These findings underscore the importance of tailored strategies, including physical activity programmes, psychosocial support, and health literacy initiatives, to enhance functional independence and quality of life among older adults.

## 1 Introduction

The demographic shifts of recent decades have been characterised by an increasingly ageing population (1). In developed countries, this demographic transition is driven by declining population growth rates and advancements in healthcare and quality of life, promoted by active ageing policies (2). These changes have precipitated profound socio-economic transformations, with significant implications for healthcare systems, public policies, and broader society. For example, the global population of older adults is projected to reach approximately 973 million by 2030, rising from 6.9 to 12.0% of the total population. This trend not only underscores the growing burden on healthcare services but also indicates a need for innovative policy responses to support an ageing society. In Europe, which accounts for 24.3% of this demographic (3), such changes pose significant challenges for long-term care and resource allocation.

In Portugal, which had a total population of approximately 10.3 million, data from the National Institute of Statistics (INE) indicate a marked increase in the older adult population, with 2,484,783 individuals aged 65 and older and 724,216 aged 80 and older recorded in 2020 (3). This represents nearly 24% of the total Portuguese population, highlighting the considerable demographic weight and growing impact of ageing on national health and social system. The ageing index is expected to nearly double, from 159 to 300 older adults for every 100 young people, by 2080 (3), underscoring the need for robust healthcare planning and sustainable policy initiatives. Moreover, despite Portugal’s strong performance in life expectancy and healthy life years, the high prevalence of any functional limitations in daily activities—affecting 60% of individuals aged 65 and over-emphasises the pressing issue of functional dependence (4).

Ageing is a complex process characterized by a gradual, dynamic, and progressive interaction of biological, psychological, and social factors that manifest uniquely in each individual (5). Although biological age alone is not a precise determinant of ageing-related changes, the likelihood of experiencing physical and cognitive impairments increases significantly with greater life expectancy (5). Conceptual models such as the Disablement Process Model (6) and the World Health Organization (WHO) International Classification of Functioning, Disability and Health (7) offer valuable insights into the development of functional dependence by suggesting that the progressive decline in functionality is influenced not only by physiological decline but also by environmental and personal factors. One of the primary challenges associated with ageing is the progressive loss of functionality, which directly affects the older adults’ ability to perform essential activities of daily living (8–10) and is closely linked to increased frailty, violence and institutionalisation. Dependency in basic activities of daily living (BADL) and instrumental activities of daily living (IADL) has direct implications for quality of life and healthcare service utilisation, serving as an important predictor of long-term care needs (8, 9, 11). Also, the high prevalence of chronic diseases exacerbates functional limitations and loss of autonomy, especially among older individuals (12).

The assessment of functional ability is a crucial step in the planning of personalised care, clinical decision-making, and the formulation of public health policies. For this reason, standardised instruments are widely used to evaluate the degree of dependency among older populations. Among the most recognised tools are the Barthel Index, which assesses autonomy in BADLs, and the Lawton and Brody Scale, which focuses on IADLs. The Barthel Index, validated in various contexts, is extensively used in geriatric research due to its simplicity, reliability, and ability to classify different levels of dependence in self-care tasks (8, 13). Meanwhile, the Lawton and Brody Instrumental Activities of Daily Living Scale enables the evaluation of independence in more complex daily tasks, such as medication management, telephone use, transportation, and financial management (10, 11, 13, 14). The scale’s sensitivity in detecting early declines in autonomy makes it particularly valuable in both community and primary care settings. In such contexts, the selection of functional assessment tools should strike a balance between feasibility with clinical validity. The combined use of the Barthel Index and the Lawton-Brody Scale constitutes an effective strategy, offering a comprehensive view of functional autonomy (13, 15, 16). These instruments are especially well-suited to the realities of primary care due to their brevity, ease of administration, and well-established psychometric properties.

The WHO underscores the importance of preventive measures and continuous monitoring of older people, with a comprehensive approach to promoting health and well-being. Although ageing can lead to limitations, especially in the presence of chronic diseases, self-care and appropriate health management can improve quality of life and reduce complications, resulting in lower healthcare costs (17). Additionally, promoting health literacy can also contribute to improved quality of life and reduced costs associated with harmful health decisions and behaviours (18). However, older adults generally exhibit lower levels of health literacy, impeding their ability to access and utilise health information effectively (18). Thus, the implementation of strategies to improve health literacy is imperative for promoting functional autonomy and reducing dependency among this population, thereby alleviating the overall strain on healthcare systems (19). The functionality of older adults is influenced by individual and contextual factors, including family support and the structure of healthcare services (20). The decrease in functionality has implications not only for the individual but also for the family, community and the sustainability of the health and social systems (21). The family’s role in caregiving is widely recognised, as family members are often the first to identify changes in the older adult’s health status and to provide emotional and practical support. However, the family’s ability to provide such support may be affected by sociodemographic changes, such as reduced family size and increased female participation in the labour market (22).

In this context, Family Health Units (FHUs) play a pivotal role in monitoring the older adult population. In Portugal, these units are part of the National Health Service’s Primary Healthcare framework and operate with a multidisciplinary team, typically composed of family doctors, nurses, and administrative staff, often complemented by other healthcare professionals such as psychologists, nutritionists, and social workers. FHUs are responsible for delivering comprehensive and continuous care to a defined population within a designated catchment area. Their core functions include regular health monitoring, chronic disease management, and the prevention of functional dependence. The proximity of these units to users, along with the therapeutic relationships established with families enable FHUs to deliver early and personalised interventions that promote healthy ageing and the maintenance of autonomy. However, there is a scarcity of studies assessing the degree of dependency among older adults monitored within these units, particularly in the Portuguese context, thereby underscoring the need for further research. Also, the WHO highlights the importance of expanding research focused on the needs of older adults as a priority for healthy ageing (23). In alignment with this approach, European Union member states have also discussed new strategies to anticipate and address the challenges posed by an ageing population, in accordance with the United Nations 2030 agenda for sustainable development (21). In the context of public policies, Portugal’s National Health Plan 2030 underscores the necessity of implementing measures to improve older adults’ quality of life and reduce the burden on healthcare services (23). A comprehensive characterisation of functional dependence among older adults can inform these policies, enabling the development of more targeted and effective interventions.

The present study aimed to assess the degree of dependency in basic and instrumental activities of daily living among older adults monitored by a Family Health Unit (FHU) in northern Portugal. Specifically, it sought to: (1) quantify levels of functional dependence; (2) examine the association between functional dependence and key sociodemographic factors (including gender, marital status, and educational level); and (3) provide empirical evidence to inform the development of tailored health intervention strategies and public policies addressing the needs of an ageing population. This article is structured as follows: Section 1 provides the background and rationale of the study; Section 2 presents the methodological approach, including sample selection and data collection tools; Section 3 details the main results; Section 4 discusses the findings in light of existing literature and outlines the implications for practice and policy, as well as the study’s limitations.

The main contribution of this study is to provide a detailed, population-based assessment of dependency in basic and instrumental activities of daily living among older adults followed by a Family Health Unit. By identifying sociodemographic predictors of autonomy, the study offers empirical evidence to guide tailored healthcare interventions and inform public policy for ageing populations.

## 2 Data and methods

### 2.1 Data

A population-based cross-sectional study with a random sample stratified by age (65–74; 75–84; and 85 or older) and gender, included 5.7% of patients aged 65 or older enrolled at a FHU in Northern Portugal. The northern region of Portugal was chosen for this study due to its notably ageing demographic profile and documented regional disparities in healthcare accessibility and support services. This area consistently shows a higher proportion of older adults, making it a strategic setting for examining functional dependency in primary care. Inclusion criteria included being enrolled at the FHU and being 65 years or older on January 1, 2023. Individuals with outdated phone contact information were excluded from the study.

As of January 1, 2023, the FHU had 3,709 enrolled patients aged 65 or older. The sample size was calculated through power analysis, based on an expected dependency rate of 29.2%, as reported in a previous study (24) on the Portuguese population, with a precision of 5% and a confidence level of 90%. The sample closely reflects the age and gender distribution of the total FHU population, with minor variations (Table 1). While the study did not specifically stratify by health status or socioeconomic factors, the FHU provides care to all enrolled individuals regardless of socioeconomic background. Therefore, the sample is expected to broadly represent the diversity of the enrolled population. Participant selection was conducted using a list of individuals aged 65 and older, provided by the institution’s planning and statistics division. A random number table was employed to select the sample. The list of potential participants was extended beyond the required sample size allowing for substitutions. If a selected participant could not be reached due to death, relocation, or other reasons, a substitute participant from the list was included to maintain the intended sample size. Table 1 shows the demographic distribution of the population aged 65 or older enrolled at the FHU and the number of participants selected by gender. The study followed the STROBE guidelines for cross-sectional studies, a set of internationally recognised recommendations that enhance the quality and transparency of observational research by providing a checklist for comprehensive reporting of study design, methods, and results (25).

**Table 1:** Population enrolled at the FHU and participants under study.

	FHU Population		Sample	
	Men	Women	Men	Women l
Age				
65–74	910	1,110	53	64
75–84	537	675	31	36
+85	155	322	9	19

Participants were invited to participate in the study through phone calls made by a member of the research team (working at the FHU) who provided information about the study, its objectives, and procedures. If the initial call was not answered, up to three further attempts additional were made on different days and at different times. If the selected participant agreed to participate, a date was scheduled for a researcher to administer the questionnaire via telephone. To minimise potential bias, interviewers underwent training to guarantee that the interviews were conducted in a clear and objective manner, ensuring participants’ comprehension of the questions. If the older adult was unable to respond, a caregiver could provide information, if consent was obtained. The interviews were conducted in the first semester of 2023.

### 2.2 Material

A sociodemographic characterisation questionnaire was developed to collect data on age, gender, marital status, and type of family. To assess autonomy in BADL, the Portuguese-validated version of the Barthel Index (24) was used. To assess autonomy in IADL, the Portuguese-validated version of the Lawton-Brody scale (26) was used.

The Barthel Index is a standardised and widely used measure for assessing functional ability in performing BADL. It quantifies an individual’s level of independence across 10 activities, providing a total score ranging from 0 to 100, with higher scores indicating greater independence. The activities assessed include feeding (eating without assistance); bathing; personal hygiene (ability to perform activities such as brushing teeth and combing hair); dressing and undressing; sphincter control (bladder); sphincter control (bowel); toilet use; transfers (from chair to bed and vice versa); mobility (ability to walk or use a wheelchair without assistance); and climbing stairs. Each activity is scored at different levels of independence, with scores varying per activity (e.g., 0, 5, 10 or 15 points). The sum of the scores is used to categorise individuals into different levels of dependency: 0–20 points (total dependency); 21–60 points (severe dependency); 61–90 points (moderate dependency); 91–99 points (mild dependency); and 100 points (total independence).

The Lawton-Brody Scale is an instrument that assesses functional ability in performing IADL. These activities are essential for maintaining independence within a community setting and include more complex tasks than BADL. The scale is particularly useful for assessing the autonomy of older adults and identifying support needs and interventions. The scale evaluates an individual’s ability in eight key domains: telephone use (dialling numbers, answering calls, and seeking information); shopping (planning and making purchases independently); meal preparation (planning, preparing, and serving meals); housekeeping (performing household chores such as cleaning and tidying); laundry; transportation use (public or private); responsibility for one’s own medication (correctly managing and taking medication); and money management (finances, bill payments, and keeping financial records). Each domain is scored based on the level of independence, ranging from zero (total dependency in performing the activity) to one point (independence in performing the activity). The total score ranges from 0 to 8 points, with higher scores indicating greater functional independence.

### 2.3 Ethical considerations

The study was conducted according to the ethical principles established in the Declaration of Helsinki and the Oviedo Convention and received approval from the Local Health Unit’s ethics committee (reference no. 111/CE/JAS, 11 December 2020). Before data collection, participants who consented to take part were reminded of the study’s objectives, procedures, and potential benefits and risks. Participation was voluntary, and participants were free to withdraw at any time without any consequences for their care at the FHU. Confidentiality was guaranteed, and participants were informed that the data would be used exclusively for research purposes. Before the interview, the informed consent form was read aloud, and participants were asked to confirm their agreement. To ensure data protection, all information was anonymised, coded, and stored in a password-protected system accessible only to the research team. Encrypted identifiers were assigned to participants, ensuring anonymity. In cases where health concerns or undiagnosed changes in health status were identified during the interviews, participants were referred to their doctor or family nurse to ensure appropriate follow-up care. These measures were implemented to uphold ethical integrity and to safeguard participants’ rights and well-being.

### 2.4 Data analysis

The data were analysed using descriptive and analytical statistical techniques. The analysis was performed using IBM SPSS Statistics, version 29.0. A descriptive analysis was conducted to characterise the sample’s sociodemographic variables (age, gender, marital status, type of family) and levels of dependency in BADL and IADL. Frequencies and percentages were used for categorical variables, while means (M) and standard deviations (SD) were used for continuous variables. The t-test for independent samples was applied to compare the means of two independent samples. ANOVA was used to compare the means of dependency in BADL and IADL among the three age groups. When deemed necessary, adjustment for multiple comparisons was performed using the Bonferroni method to control for type I error in analyses involving multiple hypotheses. Pearson’s correlation coefficient was used to study the strength of the association between two continuous variables. A hierarchical binary logistic regression was conducted to identify factors associated with autonomy in BADL and IADL. The assumptions of logistic regression were tested, including the absence of multicollinearity, which was assessed using the Variance Inflation Factor (VIF). Model fit was evaluated using the Hosmer-Lemeshow test, while predictive capacity was assessed with Nagelkerke’s R^2^. In the first block, the model included age as a continuous variable, whereas gender, marital status (widowed vs. non-widowed), and literacy (illiterate vs. other education levels) were added to the second block. Odds ratios (OR) with 95% confidence intervals (CI) were reported to quantify the strength of associations between independent variables and autonomy levels.

A *p*-values below 0.05 were considered indicative of statistically significant differences or associations. These analyses allowed for the assessment of the distribution of variables of interest, identification of significant associations, and comparison of dependency levels across different subgroups within the study population. The results are presented in the form of tables to facilitate interpretation and discussion of the findings.

## 3 Results

In addition to the list of selected participants, it was necessary to replace 12 individuals (4 refused to participate; 2 had died; 3 did not answer the phone after four attempts; 2 had been transferred to another FHU; and 1 had outdated contact details).

A total of 212 participants were included in the study (119 females and 93 males). Of these, 55.2% (117) were aged 65–74 years, 31.6% (67) were aged 75–84 years, and 13.2% (28) were 85 years or older. Table 2 summarises the sample characteristics. Most participants (64%) had completed 4 years of formal education, and 66.5% were married, with couples representing the most common type of family (55.6%).

**Table 2:** Characterisation of the study sample.

	65–74 (*n* = 117)	75–84 (*n* = 67)	+85 (*n* = 28)	Total (*N* = 212)
Gender				
Female	54.70(64)	53.73(36)	67.86(19)	56.13(119)
Male	45.30(53)	46.27(31)	32.14(9)	43.87(93)
Marital status				
Married	76.07(89)	62.69(42)	35.71(10)	66.51(141)
Single	2.56(3)	4.48(3)	3.57(1)	3.30(7)
Divorced	13.68(16)	1.49(1)	0(0)	8.02(17)
Widower	7.69(9)	31.34(21)	60.71(17)	22.17(47)
Education level				
Illiterate	0.85(1)	5.97(4)	25.00(7)	5.66(12)
Primary	59.83(70)	73.13(49)	60.71(17)	64.15(136)
Secondary	15.38(18)	11.94(8)	7.14(2)	13.21(28)
High School	8.55(10)	2.99(2)	7.14(2)	6.60(14)
Higher Education	15.38(18)	5.97(4)	0(0)	10.38(22)
Type of family				
Extended	7.69(9)	19.40(13)	25.00(7)	13.68(29)
Couple	69.23(81)	44.78(30)	25.00(7)	55.66(118)
Cohabitation	0(0)	0(0)	3.57(1)	0.47(1)
Institutional	0.85(1)	5.97(4)	14.29(4)	4.25(9)
Single-Parent	1.71(2)	2.99(2)	3.57(1)	2.36(5)
Nuclear	8.55(10)	11.94(8)	7.14(2)	9.43(20)
Single-Person	11.97(14)	14.93(10)	21.43(6)	14.15(30)
Barthel Index				
Total Dependency	0.85(1)	5.97(4)	14.29(4)	4.25(9)
Severe Dependency	0(0)	7.46(5)	14.29(4)	4.25(9)
Moderate Dependency	3.42(4)	2.99(2)	17.86(5)	5.19(11)
Mild Dependency	11.11(13)	5.97(4)	10.71(3)	9.43(20)
Independency	84.62(99)	77.61(52)	42.86(12)	76.89(163)
M(SD)	97.99(9.89)	89(25.67)	74.64(36.00)	92.17(22.01)
Lawton and Brody Scale				
Total Dependency	0.85 (1)	13.43 (9)	32.14 (9)	8.96(19)
Severe Dependency	0(0)	2.99 (2)	7.14 (2)	1.89(4)
Moderate Dependency	0.85(1)	1.49 (1)	7.14 (2)	1.89(4)
Mild Dependency	7.69(9)	16.42(11)	21.43 (6)	12.26(26)
Independency	90.60 (106)	65.67 (44)	32.14 (9)	75.00(159)
M(SD)	7.57(1.14)	6.12(2.62)	3.96(3.12)	6.64(2.37)

### 3.1 Functional independence in BADL and IADL

As shown in Table 2, 76.89% (163) of participants were fully independent in BADL, while 9.44% (20) exhibited mild dependence. For IADL, 75.00% (159) were fully independent, while 12.27% (26) had a mild dependence.

The analysis of variance for the Barthel Index by age group revealed significant differences [*F* (2,209) = 15.25; *p* < 0.001], with differences observed between all three age groups. Similarly, differences were found in mean scores for the Lawton and Brody Scale [*F* (2,209) = 36.66; *p* < 0.001]. Results showed a negative linear correlation between age and independence in BADL (*r* = −0.41) and IADL (*r* = −0.56).

The prevalence of dependency in BADL and IADL increased with age, being significantly higher among individuals aged 85 or older. A clear progression of functional dependence was observed across the analysed age groups, as shown in Table 3. It is estimated that between 21.75 and 24.47% of the population enrolled at the FHU experience dependency in performing BADL, while between 23.61 and 26.39% experience dependency in performing IADL. These estimates are particularly concerning for the participants aged 85 years or older, with 52.70 to 61.58% and 63.66 to 72.04% showing impairment in performing BADL and IADL, respectively. Total dependency in BADL was rare (0.9% of the sample), whereas total dependency in IADL was more common (7.7%).

**Table 3:** Prevalence of dependency in BADL and IADL by age group and associated confidence intervals.

Age	Population	% Dependency in BADL	IC95%	% Dependency in IADL	IC95%
65–74	2020	15.38	13.81–16.95	9.40	8.13–10.67
75–84	1,212	22.39	20.04–24.74	34.33	31.66–37.00
+85	477	57.14	52.70–61.58	67.85	63.66–72.04
Global	3,709	23.11	21.75–24.47	25.00	23.61–26.39

### 3.2 Factors associated with autonomy in BADL and IADL

A hierarchical binary logistic regression was conducted to examine whether age, gender, marital status and literacy predicted autonomy in BAD and IADL. The predictors were entered sequentially in two steps: in the first step, age as a continuous variable was entered; in the second step, gender, marital status (widowed vs. non-widowed) and literacy (illiterate vs. other educational levels) were added to the model. The model for BADL was statistically significant*, χ*^2^(3) = 8.754, *p* = 0.033, explaining 21.5% of the variance (Nagelkerke R^2^ = 0.215). Similarly, the model for IADL was also statistically significant, *χ*^2^(3) = 14.520, *p* = 0.002, explaining 40.0% of the variance (Nagelkerke R^2^ = 0.400). The analysis revealed that age and gender were significant predictors for both dimensions of autonomy, while marital status (widowed) showed a significant association only with autonomy in IADL, as demonstrated in Table 4. For BADL, age showed a significant association with autonomy (β = 0.095, *p* < 0.001), indicating that for each additional year of age, the odds ratio of being dependent increased by 10% (OR = 1.100; 95% CI: 1.046–1.157). Gender was also a significant predictor (β = 1.049, *p* = 0.012), with women having 2.85 times higher odds of dependency in BADL (OR = 2.854, 95% CI: 1.261–6.456). However, neither marital status nor literacy demonstrated statistically significant associations with autonomy in BADL.

**Table 4:** Factors associated with autonomy in BADL and IADL: results of the hierarchical binary logistic regression.

	β	S.E.	Wald	df	Sig.	Exp(B)	95% C.I. for EXP(B)		VIF
							Lower	Upper	
BADL									
Age	0.095	0.026	13.598	1	<0.001	1.100	1.046	1.157	1.332
Gender	1.049	0.417	6.337	1	0.012	2.854	1.261	6.456	1.156
Widowhood	0.114	0.462	0.061	1	0.805	1.120	0.453	2.769	1.425
Illiteracy	0.017	0.686	0.001	1	0.980	1.017	0.265	3.899	1.148
Constant	−9.171	2.013	20.759	1	<0.001	0.000			
IADL									
Age	0.138	0.029	22.508	1	<0.001	1.148	1.084	1.216	1.332
Gender	0.877	0.439	3.995	1	0.046	2.405	1.017	5.686	1.156
Widowhood	0.983	0.459	4.593	1	0.032	2.674	1.088	6.572	1.425
Illiteracy	0.160	0.774	0.043	1	0.836	1.174	0.257	5.351	1.148
Constant	−12.510	2.296	29.681	1	<0.001	0.000			

For IADL, age demonstrated a significant association, indicating that for each additional year of life, the odds ratio of being dependent in IADL increased by 14.8% (β = 0.138, *p* < 0.001, OR = 1.148, 95% CI: 1.084–1.216), confirming the trend of increasing dependency with ageing.

Gender also retained a significant association (β = 0.877, *p* = 0.046, OR = 2.405, 95% CI: 1.017–5.686) with women showing 2.41 times higher odds of dependency. Additionally, being widowed was associated with reduced autonomy in IADL (β = 0.983, *p* = 0.032, OR = 2.674, 95% CI: 1.088–6.572). However, as observed in BADL, illiteracy was not a significant predictive factor for IADL (*p* = 0.836). Overall, women tend to exhibit higher levels of dependency in both BADL and IADL. Age showed a negative correlation with autonomy in both dimensions, being a significant predictive factor for functional dependence.

## 4 Discussion and conclusion

This study aimed to examine the dependency profile of individuals aged 65 and older enrolled at a FHU regarding their performance in BADL and IADL. The sociodemographic characteristics of the participants align with the demographic profile of the Portuguese population. The higher prevalence of women in the older age groups reflects the increased life expectancy and the feminisation of ageing, a phenomenon attributed to women’s generally lower exposure to certain risk factors compared to men (27, 28).

The prevalence of older adults with impairments in BADLs reported in population-based studies varies according to the context and assessment instruments used. For example, a study in Brazil (29) found that 8.1% of older adults had impairments in at least one BADL, while another reported a higher percentage of 17% (9). A study conducted in China showed that 19.02% of older adults had impaired autonomy in BADLs (8). Similarly, an epidemiological study in Poland, using the Katz Index, revealed that 17.30% of older adults had impairments in BADL (14). Notably, all studies used the criterion of individuals aged 60 years or older (8, 9, 14, 29). In contrast, one study based on national data and using the Lawton and Brody Daily Living Index found that 11.02% of the individuals aged 65 and over had impairments in at least one BADL (10). However, the present study identified a higher prevalence (23.1%), although this percentage might be explained by the fact that the sample was drawn from a FHU population rather than the general community. Regarding dependency in IADLs, the present study reports lower values compared to the studies mentioned above (8, 10, 14, 29), except for one study which reported a value of 23% (9), while the value found in the present study was 25%.

Women showed greater dependency in both BADL and IADL, consistent with findings from previous studies (9, 10, 30). Women tend to live longer than men, placing them at higher risk of developing chronic and debilitating conditions that impair functional abilities (31). Greater longevity means that women are more likely to experience age-related declines in physical and cognitive abilities. Additionally, women are more susceptible to certain chronic conditions, such as arthritis, osteoporosis, and autoimmune diseases, which can hinder mobility and the ability to perform daily activities independently (31). These conditions often directly impact the ability to perform BADL and IADL (14).

Older women are more likely to experience widowhood and social isolation due to their greater life expectancy. The loss of a partner and reduced social interactions can increase the risk of depression and anxiety, which can further impair functional abilities (10, 32). Historically, women have had fewer opportunities to engage in regular physical activities compared to men (13). A lifetime of insufficient physical activity can lead to reduced muscle mass and increased frailty in old age, ultimately heightening dependency (33, 34). Moreover, many women dedicate much of their adult lives to caring for others, such as raising children or caring for older adult relatives, often neglecting their health. This dedication to the caregiver role can lead to a lack of preparedness for self-care in old age, contributing to greater dependency (35). Older women are also more affected by economic barriers, such as lower incomes and reduced access to healthcare resources (36). Lower education attainment, frequently observed in many cases, may further limit knowledge and access to effective self-care practices and support resources, thus exacerbating dependency (37, 38). In contrast, older men are more likely to have spouses or partners caring for them, while many older women live alone after widowhood (35). Although the present study did not establish literacy as a predictor of dependency, existing literature suggests that low education levels are associated with functional incapacity (39), with illiterate older adults exhibiting greater dependency in both basic and instrumental activities. Low educational attainment has been associated with a higher risk of disability and mortality, mainly because learning opportunities can help people develop skills and self-confidence to adapt and pursue healthier ageing processes (40). However, this discrepancy may be explained by several factors. First, measurement limitations could have influenced this result, as literacy was self-reported, potentially leading to classification bias. Second, the study may have had limited statistical power to detect a significant association due to the sample size. Lastly, cultural and social factors specific to the study population may play a role. In Portugal, strong family and community support networks may mitigate the impact of illiteracy on functional capacity, allowing older adults to maintain independence despite lower education levels.

An important finding of the present study was assessing and estimating functional autonomy as a function of age in the population of the FHU. The results indicate that with each additional year of life, the risk of dependency in the BADL increases by 10%, meaning that, over a decade, the probability of losing autonomy nearly doubles. For IADL, the impact of ageing is even more pronounced, with a 14.8% annual increase in the risk of dependency. This finding underscores a more rapid decline in autonomy for complex tasks, such as financial management or using public transportation. Being a woman increases the risk of dependency in ADL by 65% compared to men, indicating greater vulnerability in women to the loss of autonomy in basic activities such as personal hygiene, dressing, or eating. This evidence is substantially greater in IADL, with women facing more than twice the risk of dependency as men. This suggests greater difficulty in performing instrumental activities, such as financial management, transportation, or using technology, reflecting a more significant impairment of functional autonomy in this domain. These data suggest that women face greater challenges regarding functional dependence as they age (10, 29, 32, 35, 41, 42). The inability of older adults to be self-sufficient in performing basic and instrumental tasks or managing self-care poses complex challenges for family support networks and healthcare professionals (30).

### 4.1 Implications for practice

Understanding the burden of dependency within the community is essential for developing comprehensive strategies and effective interventions to mitigate functional decline in older adults, particularly among women (43). The findings of this study highlight the urgent need for proactive and multidisciplinary strategies to address functional dependence in BADL and IADL. A key intervention is the development of structured physical activity programmes tailored to older adults. These programmes should incorporate strength and flexibility exercises in community centres and gyms, with specialized training of fitness instructors and physiotherapists working with the older adult population (44, 45). To maximize adherence, awareness campaigns and financial incentives should be implemented, particularly targeting older women, who exhibited higher dependency levels in this study.

Beyond physical health, it is crucial to reinforce psychosocial support mechanisms. The findings of this study suggest that widowhood and social isolation exacerbate the risk of dependency, underscoring the importance of community-based mental health interventions. Establishing peer support groups, psychological counselling services, cognitive training programmes, and volunteer mentorship initiatives can provide critical emotional and social support (46, 47). Additionally, organizing community engagement events can foster social participation, promoting meaningful interactions and reducing isolation (47).

Health literacy plays a fundamental role in maintaining autonomy (17). Therefore, expanding health education and self-care training through structured workshops, courses, and educational materials focused on managing chronic diseases, nutrition, and overall well-being, is crucial (17, 48). The integration of accessible digital technologies, such as telehealth services and telephone helplines, can facilitate access to critical information and remote support, particularly for those with mobility limitations (49).

Financial constraints significantly impact dependency among older adults, highlighting the need for comprehensive support policies. Partnerships with local government institutions can facilitate access to existing programmes and resources. Enhanced retirement benefits and health subsidies would enable older adults to access the necessary healthcare services and assistive devices. Additionally, targeted financial assistance programmes should be developed to help cover medical expenses and facilitate the acquisition of essential equipment that promotes independence in daily activities.

Finally, strengthening community and family support networks is paramount. Family caregivers play a central role in supporting older adults, yet they frequently lack adequate training and respite opportunities (22). Developing caregiver education programmes and implementing respite care services can improve the quality of informal care while ensuring caregivers’ well-being (36).

### 4.2 Study limitations

This study was limited to a single FHU, hindering the generalisability of findings. Its cross-sectional design, with data collection at a single moment in time, limits the ability to establish cause-and-effect relationships between variables. Although the sample was randomly selected and stratified by age and gender, it accounted for only 5.7% of the patients enrolled at the FHU. The small proportion of participants may not fully capture the diversity of the older adult population in the region, thus limiting the generalisability of results to other contexts. Also, data collection was conducted by telephone interviews, relying on the accuracy and truthfulness of the participants’ or their caregivers’ responses. Potential memory biases or misunderstandings of the questions may have affected data quality. Furthermore, the study did not include data on potential confounding factors such as chronic diseases, economic status, social support, or access to healthcare services, all of which could influence dependency levels in BADL and IADL. The exclusion of these variables limits a more in-depth analysis of the interactions between these factors and the outcomes. Therefore, the study results should be interpreted with caution. Future studies could benefit from adopting a longitudinal design, incorporating larger samples, and more diverse data collection methods to improve the validity and generalisability of the findings.

The study provided valuable insights into the level of dependency in BADL and IADL among older adults enrolled in a FHU in the north of Portugal. By identifying patterns of functional dependence and providing support for planning health actions, this study contributes to improving the quality of life and functional autonomy of the population under study.

The results indicate a higher prevalence of functional dependence and frailty among women, particularly after the age of 75, highlighting the importance of targeted intervention strategies to promote autonomy and quality of life for older adults. The findings underscore the need to implement comprehensive programmes to promote functional independence among older adults, especially women. These study findings also highlight the importance of programmes for physical activity, psychological and social support, health education, and improvements in economic benefits and support networks. At the local level, FHUs should prioritize the development and implementation of multidisciplinary programmes that include physical activity, psychological and social support, and health education, while also advocating for improved economic benefits and support networks (25).

## Data Availability

The raw data supporting the conclusions of this article will be made available by the authors, without undue reservation.
